# Evolutionary Association of Stomatal Traits with Leaf Vein Density in *Paphiopedilum*, Orchidaceae

**DOI:** 10.1371/journal.pone.0040080

**Published:** 2012-06-29

**Authors:** Shi-Bao Zhang, Zhi-Jie Guan, Mei Sun, Juan-Juan Zhang, Kun-Fang Cao, Hong Hu

**Affiliations:** 1 Key Laboratory of Economic Plants and Biotechnology, Kunming Institute of Botany, Chinese Academy of Sciences, Kunming, Yunnan, China; 2 Key Laboratory of Tropical Plant Ecology, Xishuangbanna Tropical Botanical Garden, Chinese Academy of Sciences, Kunming, Yunnan, China; 3 State Key Laboratory of Plant Physiology and Biochemistry and College of Agronomy and Biotechnology, China Agricultural University, Beijing, China; CNR, Italy

## Abstract

**Background:**

Both leaf attributes and stomatal traits are linked to water economy in land plants. However, it is unclear whether these two components are associated evolutionarily.

**Methodology/Principal Findings:**

In characterizing the possible effect of phylogeny on leaf attributes and stomatal traits, we hypothesized that a correlated evolution exists between the two. Using a phylogenetic comparative method, we analyzed 14 leaf attributes and stomatal traits for 17 species in *Paphiopedilum*. Stomatal length (SL), stomatal area (SA), upper cuticular thickness (UCT), and total cuticular thickness (TCT) showed strong phylogenetic conservatism whereas stomatal density (SD) and stomatal index (SI) were significantly convergent. Leaf vein density was correlated with SL and SD whether or not phylogeny was considered. The lower epidermal thickness (LET) was correlated positively with SL, SA, and stomatal width but negatively with SD when phylogeny was not considered. When this phylogenetic influence was factored in, only the significant correlation between SL and LET remained.

**Conclusion/Significance:**

Our results support the hypothesis for correlated evolution between stomatal traits and vein density in *Paphiopedilum*. However, they do not provide evidence for an evolutionary association between stomata and leaf thickness. These findings lend insight into the evolution of traits related to water economy for orchids under natural selection.

## Introduction

Plants often exhibit considerable variations in their functional traits that affect the capture and utilization of resources and enable them to adapt to changing environments [1], [2]. The development of leaf cuticles and stomata might be linked to the success of terrestrial plants because they resolve two conflicting physiological requirements: increasing CO_2_ uptake *vs*. reducing water loss [3], [4]. Much of the evolutionary history of land plants involves leaf activities for obtaining water and preventing transpirational water losses, thereby improving their photosynthetic carbon gain and survival in dry habitats [5]. Both environment and evolutionary history are important to shape the hydraulic properties that determine how plants respond to water shortages [6]. Evolutionary pressures that drive such conservation strategies favor the coupling of the cuticle with the development of stomata [7]. Consequently, one might expect a correlated evolution between leaf attributes and stomatal traits [8]. However, little work has been done on such coordination within an evolutionary context even though one could gain valuable insights into ecological and evolutionary principles [8], [9].

Water is transpired from the leaf surface through either the outer epidermal cell walls or the stomata. Although cuticles can reduce water loss from the leaf to the atmosphere, they also slow the CO_2_ diffusion in the reverse direction [10]. Therefore, stomata can effectively regulate gas exchange where water vapor leaves the plant and CO_2_ enters. The potential transpirational demand is primarily determined by both stomatal aperture and density [11]. Over time, stomata have changed markedly in their size and numbers since first appearing on the leaf surface approximately 411 million years ago [12]. Stomatal density (SD) is negatively correlated with atmospheric CO_2_ concentration, while size is positively correlated [3], [13], [14]. Although the level of atmospheric CO_2_ is a main selective agent, SD is also related to water availability, light intensity, and temperature [13], [15], [16], [17]. Water deficits lead to more densely packed but smaller stomata [17], [18]. The efficiency with which CO_2_ is taken up and water loss restricted appears to be partially a function of stomatal size [19], [20]. Small stomata enable the leaf to attain high and rapid diffusive conductance under favourable conditions, and they afford greater water-use efficiency (WUE) in dry habitats because they can react more quickly to environmental stimuli [14]. By contrast, large stomata are slower to close. Although they are less able to prevent hydraulic dysfunction in dry habitats, this lag in response may be advantageous in cool, moist, or shaded environments [19], [20].

Leaf venation provides mechanical support and carboxylate transport, and aids in replacing the water transpired during photosynthesis [21], [22]. Vein density (VD) is correlated with SD, maximum hydraulic conductance, maximum photosynthetic rate, and WUE [11], . Vein patterns are highly diverse across species, and have a significant phylogenetic signal [5], [24],[25]. Historically, the evolution of VD resulted in high photosynthetic capacity during early angiosperm diversification, and promoted species diversity among angiosperms [5]. This feature can also serve as an environmental proxy [24]. For example, Dunbar-Co *et al*. have found that Hawaiian *Plantago* taxa in drier regions have higher VD values [9]. Loss of hydraulic conductance is accompanied by stomatal closure under water deficits [26]. The density of major veins plays a role in determining leaf drought tolerance [27].

Leaf structural traits determine how plants adapt to changes in water availability [15], [28]. For example, gametophyte morphology can influence water-holding capacity in ferns [29]. A leaf with a high mass per unit area is better able to store water and maintain more stable hydraulic functioning during droughty periods [30]. Consequently, leaf thickness tends to increase with site aridity [18], [28], [31]. The potential transpirational demand by plants is primarily determined by stomata. However, when water is severely limited and the stomata reach their minimum aperture, water loss from a leaf is mainly determined by epidermal conductance [32]. The cuticle is a hydrophobic and flexible membrane composed of cutin and associated solvent-soluble lipids. One of its functions is to protect against water loss from the leaf interior [33]. Cuticular property is often correlated with transpirational demand [33], [34]. Although a thick cuticle can help prevent water loss when moisture is limited [28], [35], thickness alone is not a good predictor of a species’ drought tolerance because it is not always correlated with cuticular water permeability [4], [36].

Leaf structure can also reflect the plant response to environmental stresses, such as a low supply of soil nutrients. Evolutionary pressures usually favour investment toward chemical and structural defences in stressed plants [31]. This drought response is often similar to that for nutrient limitations, i.e., the production of small leaves with thick cuticles [31], [37]. In fact, the thickened cuticles of sclerophylls can serve as a sink for excess photosynthate because those membranes do not require phosphorus or nitrogen to form cutin, suberin, and waxes [38]. Consequently, the sclerophyll protects against leaf herbivory and abiotic physical damage [37].

The well-known genus *Paphiopedilum* within Orchidaceae comprises 66 species, with plants usually occurring in limestone or mountainous forests of tropical and subtropical zones from Asia to the Pacific islands [39]. These species vary in their growing environments, developmental habit, and leaf morphology. The low capacity for water storage in the shallow soil layer of karst areas limits water supplies. Plants in this genus manifest three contrasting growth habits: terrestrial, facultative epiphytic or obligatory epiphytic. For epiphyte species, the amount of available moisture is a factor in determining the best sites for growth. Although periodic water deficit is a main environmental stressor that limits plant growth and survival within that genus [2], some species can adapt to relatively dry, calcareous regions [40]. Drought tolerance by *Paphiopedilum* is linked to leaf anatomy [2], which is evergreen and fleshy, with distinct epidermal cuticles, but no guard cell chloroplasts [2], [40]. This lack of guard cell chloroplasts slows the induction of photosynthesis, and is considered an ecophysiological adaptation to water shortage [41], [42]. Therefore, the wide range of morphological and ecological variations among *Paphiopedilum* species provides a valuable research system for understanding morphological evolution related to water-use traits [2], [42].

Plants adapt to challenging conditions through simultaneous configurations of multiple traits [9]. Their leaf vein network, stomatal design, leaf structure and cuticle are ordinately linked to water transport, regulation, storage and conservation, respectively. Here, we investigated the stomatal traits and leaf attributes of 17 species in *Paphiopedilum* when all plants were tested in the same growing environment. Our objectives were to assess the effect of phylogeny on leaf structure and stomatal traits, and to examine any correlated evolution between them. Because the responsiveness to environmental changes is generally more similar among closely related species than among those more distantly related, we expected that stomatal traits would manifest a correlated evolution with leaf attributes.

**Table 1:** Leaf carbon stable isotope ratios (δ^13^C) and stomatal traits of 17 *Paphiopedilum* species.

Species	Growth habit	δ^13^C	SL	SW	SA	SD	SI
*malipoense*	facultative	–27.24±0.05	73.43±0.88	63.74±0.57	3681.7±64.9	17.41±1.17	11.67±0.59
*emersonii*	facultative	–23.93±0.07	56.39±0.42	53.93±0.66	2391.7±39.0	34.06±1.47	12.45±0.53
*micranthum*	facultative	–27.53±0.02	56.23±0.47	46.76±0.50	2066.9±30.7	27.57±1.31	11.48±0.52
*armeniacum*	facultative	–26.52±0.09	63.64±0.56	53.65±0.52	2686.6±42.6	29.14±1.99	13.47±0.72
*bellatulum*	facultative	–26.89±0.01	48.73±0.59	47.30±0.52	1815.8±37. 0	40.87±2.16	16.81±0.55
*concolor*	facultative	–26.60±0.08	50.14±0.61	45.58±0.59	1800.4±40.2	37.47±1.55	16.64±0.57
*hirsutissimum*	facultative	–23.32±0.16	54.27±0.64	45.22±0.44	1927.2±28.9	38.23±1.61	13.65±0.56
*tigrinum*	terrestrial	–24.00±0.07	58.06±0.54	49.24±0.85	2249.5±49.8	37.47±1.33	16.26±0.50
*henryanum*	facultative	–24.32±0.12	56.16±0.58	50.72±0.63	2243.3±43.5	55.26±2.03	18.97±0.56
*charlesworthii*	epiphytic	–26.06±0.03	49.79±0.43	43.74±0.52	1711.2±25.9	55.25±2.11	16.56±0.56
*villosum*	epiphytic	–25.32±0.03	57.70±0.57	49.95±0.52	2268.3±38.8	48.82±2.27	18.92±0.74
*gratrixianum*	facultative	–24.02±0.07	54.61±1.05	50.97±0.66	2204.3±65.7	66.23±2.46	20.53±0.72
*insigne*	terrestrial	–23.42±0.04	56.56±1.36	46.19±0.60	2054.8±60.4	34.82±1.65	15.32±0.61
*dianthum*	epiphytic	–25.12±0.08	62.10±1.43	63.63±0.67	3113.3±87.2	38.23±1.61	19.57±0.61
*wardii*	terrestrial	–24.64±0.02	71.11±0.84	54.12±0.57	3017.7±39.7	21.19±1.30	13.02±0.72
*appletonianum*	terrestrial	–24.42±0.10	77.95±0.55	57.10±1.26	3497.7±81.6	18.54±1.03	15.15±0.79
*purpuratum*	terrestrial	–23.52±0.03	68.86±0.72	59.68±0.38	3225.3±35.4	17.03±0.96	10.91±0.57

SL, stomatal length (µm); SW, stomatal width (µm); SA, stomatal area (µm); SD, stomatal density (number mm^−2^); SI, stomatal index (%).

**Table 2:** Leaf structural traits of 17 *Paphiopedilum* species. LMA, leaf mass per unit area (g m^−2^).

Species	LMA	UET	UCT	LET	LCT	MT	LT	LA	VD
*malipoense*	116.2±5.1	258.8±12.0	23.88±0.69	84.27±4.42	15.22±0.47	466.0±22.6	847.2±35.7	44.82±5.35	0.919±0.049
*emersonii*	181.9±10.3	295.0±7.3	22.50±0.61	77.12±1.64	15.56±0.81	734.2±22.2	1144.4±21.7	40.84±3.92	0.880±0.040
*micranthum*	165.8±3.7	162.8±5.1	25.87±1.14	70.14±1.64	12.32±0.68	927.4±21.2	1198.5±20.9	20.13±2.02	1.186±0.057
*armeniacum*	139.3±3.1	285.7±8.1	24.09±0.65	81.54±2.87	15.15±0.50	561.1±16.1	967.6±15.0	21.41±1.65	1.183±0.042
*bellatulum*	130.2±6.2	553.9±11.7	24.78±1.16	59.18±1.60	13.74±0.75	560.7±13.0	1212.3±21.9	18.42±1.91	1.063±0.145
*concolor*	116.8±7.4	455.9±8.3	23.04±2.02	66.88±1.73	12.38±0.78	601.0±26.1	1159.2±25.8	18.47±1.30	1.207±0.099
*hirsutissimum*	157.9±7.2	304.3±6.3	13.42±0.51	67.49±2.28	9.86±0.60	492.0±27.1	887.1±29.3	39.03±1.83	1.328±0.037
*tigrinum*	107.3±6.8	206.6±18.2	21.30±0.95	51.99±1.61	12.38±0.65	348.2±7.3	640.5±12.7	45.31±4.17	1.225±0.045
*henryanum*	139.7±12.1	271.2±17.4	23.18±0.95	61.28±2.06	14.74±0.41	586.6±39.8	957.0±58.6	30.69±1.34	1.213±0.065
*charlesworthii*	125.5±3.8	374.9±32.9	18.59±0.67	43.81±1.40	13.11±0.39	428.8±15.4	879.2±39.5	16.43±2.68	1.496±0.046
*villosum*	121.5±12.5	149.4±6.1	12.82±0.68	69.38±2.02	10.68±0.38	393.6±10.1	635.9±17.1	64.48±7.20	1.195±0.068
*gratrixianum*	115.1±4.9	104.7±3.3	14.47±0.62	55.46±1.50	10.13±0.69	513.6±5.1	698.4±5.8	41.35±6.34	1.191±0.071
*insigne*	134.1±6.0	198.5±7.7	12.46±0.50	59.00±2.02	10.68±0.53	558.7±16.1	839.2±16.8	44.04±2.53	1.020±0.058
*dianthum*	237.4±15.1	606.2±46.3	24.69±1.88	66.46±1.50	12.41±0.87	824.1±40.7	1533.8±71.4	74.91±5.89	0.971±0.038
*wardii*	100.8±1.9	246.9±9.2	16.11±0.62	71.25±3.63	11.77±0.57	405.4±12.8	751.4±19.6	26.89±1.34	0.796±0.047
*appletonianum*	138.4±15.8	241.2±6.0	14.28±0.56	91.54±2.80	11.86±0.56	522.4±29.6	881.3±34.2	33.76±3.38	0.651±0.031
*purpuratum*	97.0±3.8	318.5±16.4	17.57±0.55	57.08±1.12	12.37±0.64	512.8±27.9	918.2±39.1	26.82±2.28	0.628±0.033

UET, upper epidermal thickness (µm); UCT, upper cuticle thickness (µm); LET, lower epidermal thickness (µm); LCT, lower cuticle thickness (µm), MT, mesophyll thickness (µm); LT, leaf thickness (µm); LA, leaf area (cm^−2^); VD, vein density (mm mm^−2^).

**Figure 1:**
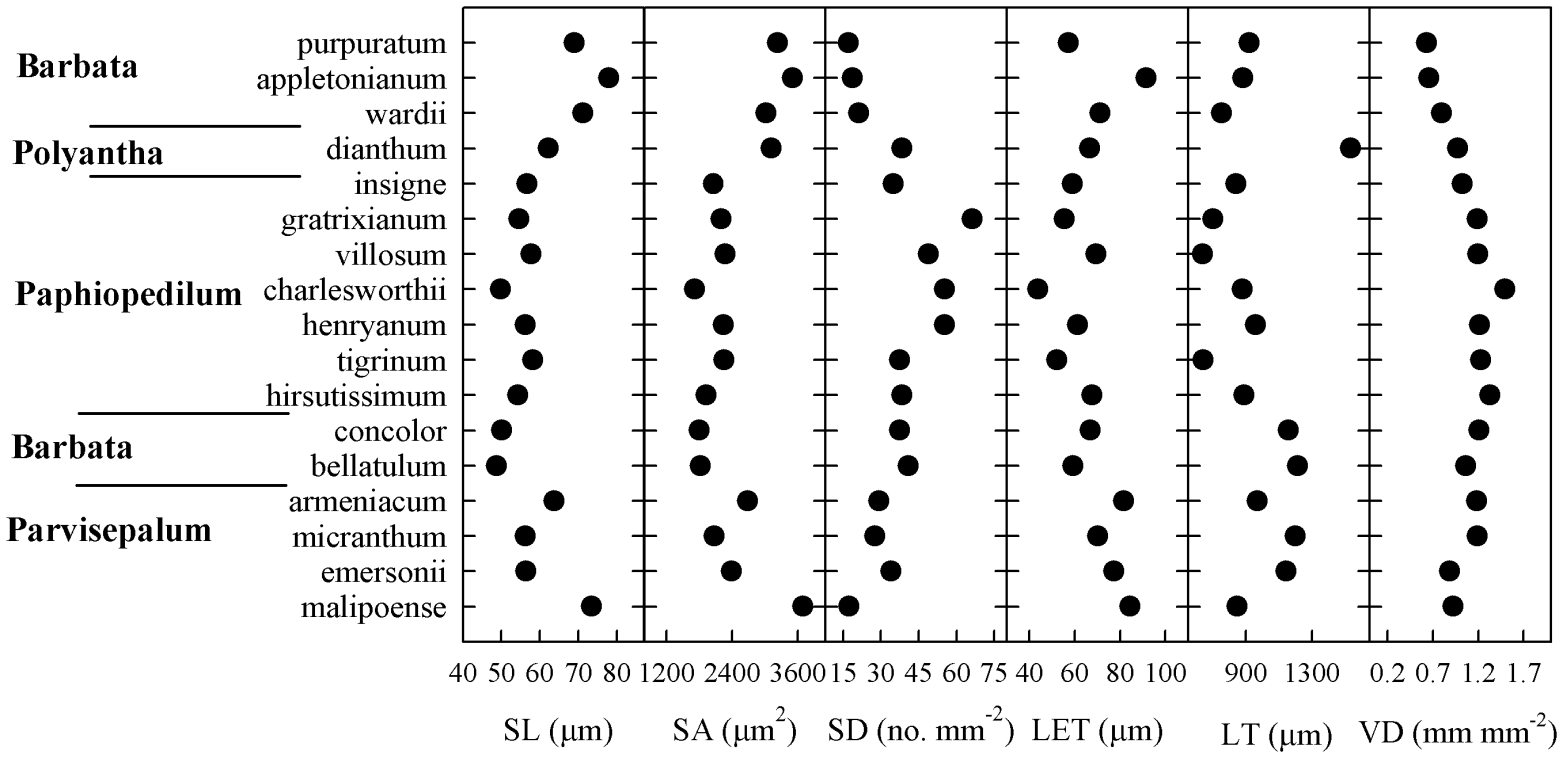
Values for leaf traits and stomatal straits in *Paphiopedilum* species. SL, stomatal length; SA, stomatal area; SD, stomatal density; LET, lower epidermal thickness; LT, leaf thickness; and VD, vein density. Names of subgenera are at left, and are based upon nuclear rDNA ITS trees from Cox *et al*. [46].

**Figure 2:**
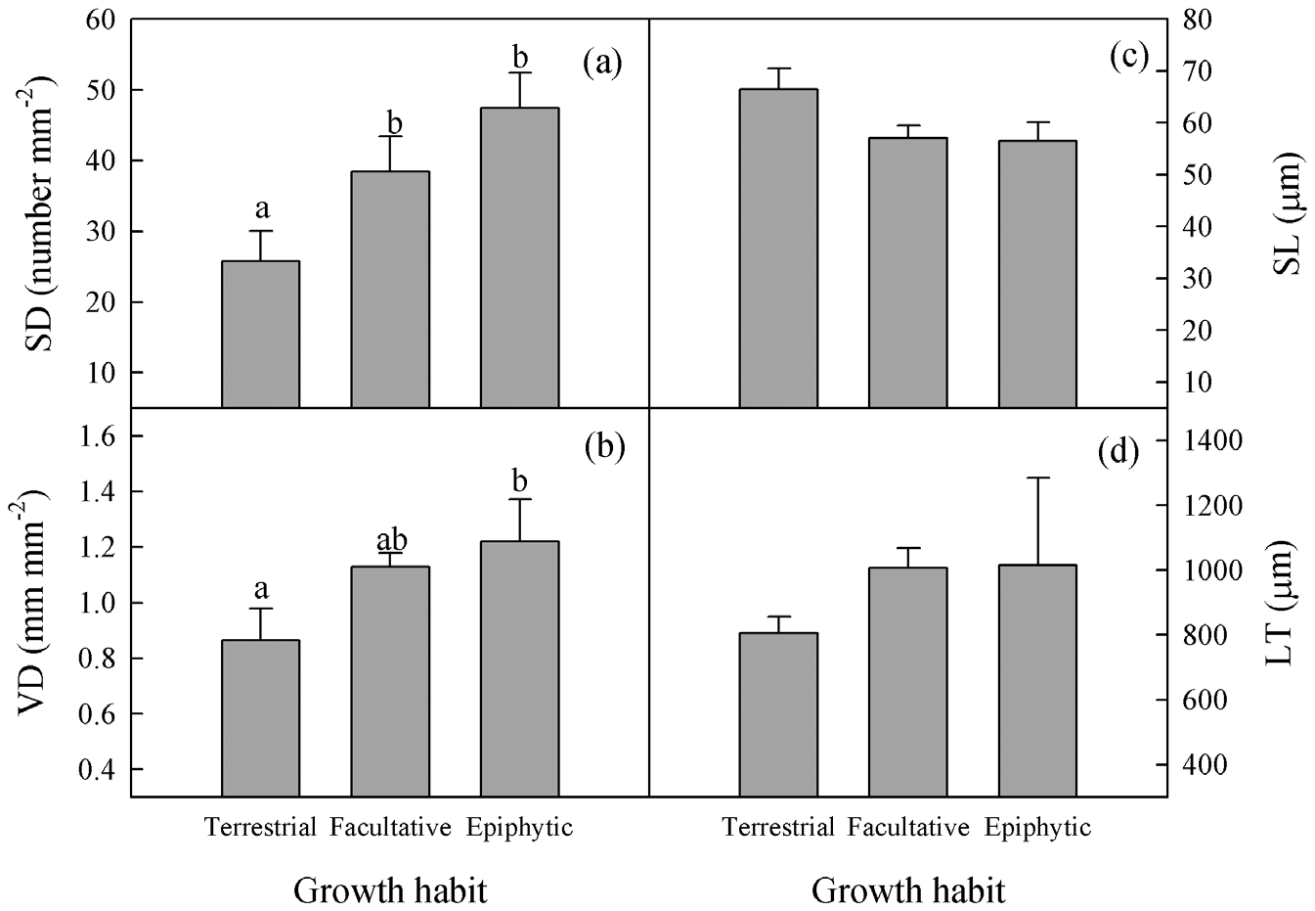
Differences in stomatal traits and leaf thickness of *Paphiopedilum* due to growth habit. SD, stomatal density; VD, vein density; SL, stomatal length; and LT, leaf thickness. Different letters above bars for each component indicate statistically different mean values (*p*≤0.05), as determined by LSD multiple comparison tests.

Phylogenetic signals of SL, SA, UCT, and TCT were >1.0, demonstrating that these traits were phylogenetically conserved (Table 3). However, the *K* values for SD and SI were <0.5, indicating that these *Paphiopedilum* relatives resembled each other less than expected, under the Brownian model, along the phylogenetic tree. These results were confirmed by our phylogenetic distribution (Fig. 1).

## Materials and Methods

### Ethics Statement

None of these experimental materials was collected from national parks or other protected areas. No tested species are under first- or second-class state protection, and they are not listed in the Inventory of Rare and Endangered Plants of China (http://zrbhq.forestry.gov.cn/portal/zrbh/s/3053/content-457748.html), or the Key Protected Inventory of Wild Plants of China (http://zrbhq.forestry.gov.cn/uploadfile/zrbh/2010-10/file/2010-10-14-bb296addeaa047798d6b6c476aaa1da9.doc). These plants were used for only scientific research as permitted by the Wildlife Protection and Administration Office under the Forestry Department of Yunnan Province.

### Plant Materials

Sample plants representing 17 species of *Paphiopedilum* were collected from their natural habitats and grown in a greenhouse at Kunming Institute of Botany, CAS (elev. 1990 m, E102°41′, N25°01′). Applying similar culturing practices largely helped to minimize any plastic differences among species in functional traits that might have resulted from environmental heterogeneity. Thus, any variations would likely reflect the role of a genetic component. Conditions included 30 to 40% of full sunlight controlled by shade nets and an ambient temperature of 20 to 25°C. Before the sample plants were analyzed, these plants were watered as needed, and were then cultivated for two to three years to ensure that their adaptation to a new environment was complete.

**Table 3:** Phylogenetic signal (*K*) of leaf attributes and stomatal traits in 17 *Paphiopedilum* species.

	*K*	*p*
SL	1.215	0.001
SW	0.761	0.010
SA	1.078	0.001
SD	0.199	0.796
SI	0.283	0.573
UET	0.836	0.017
UCT	1.100	0.004
PTT	0.626	0.082
STT	0.547	0.118
LET	0.582	0.066
LCT	0.771	0.018
MT	0.532	0.161
LT	0.772	0.019
TCT	1.207	0.004
LMA	0.723	0.037
LA	0.569	0.068
VD	0.729	0.016

*K* <1 indicate that relatives resemble each other less than expected under Brownian motion evolution along the phylogenetic tree; while *K* >1 show that close relatives are more similar than expected. SL, stomatal length; SW, stomatal width; SA, stomatal area; SD, stomatal density; SI, stomatal index; UET, upper epidermal thickness; UCT, upper cuticular thickness; PTT, palisade tissue thickness; STT, spongy tissue thickness; LET, lower epidermal thickness; LCT, lower cuticular thickness, MT, mesophyll thickness; LT, leaf thickness; TCT, total cuticular thickness; LMA, leaf mass per unit area; LA, leaf area; and VD, vein density.

### Leaf Attributes

Six mature, undamaged leaves were evaluated from individual plants of each species. Leaf area (LA) was measured with a Li-Cor 3000A area meter (Li-Cor Inc., Lincoln, NE, USA). Each leaf was then divided along the midrib. One half was re-measured with the area meter, then oven-dried at 70°C for 48 h to obtain its dry weight. Specific leaf weight was expressed as leaf dry mass per unit area (LMA). The other half was cleaned for 1 h in a 5% NaOH aqueous solution. Three sections of leaf lamina were excised from the top, middle, and bottom portions, stained with 1% safranin, and mounted in glycerol to obtain the vein density (VD). Samples were photographed at 10× magnification with an Olympus U-CMAD3 light microscope (Olympus Inc., Tokyo, Japan). Vein lengths were determined from digital images via the IMAGEJ program (http://rsb.info.nih.gov/ij/). Values for VD were recorded as vein length per unit area (mm mm^−2^). Leaf stable carbon isotope ratio (δ^13^C) was analyzed using an IsoPrime100 isotope ratio mass spectrometer (Isoprime Ltd., Cheadle Hulme, UK).

**Figure 3:**
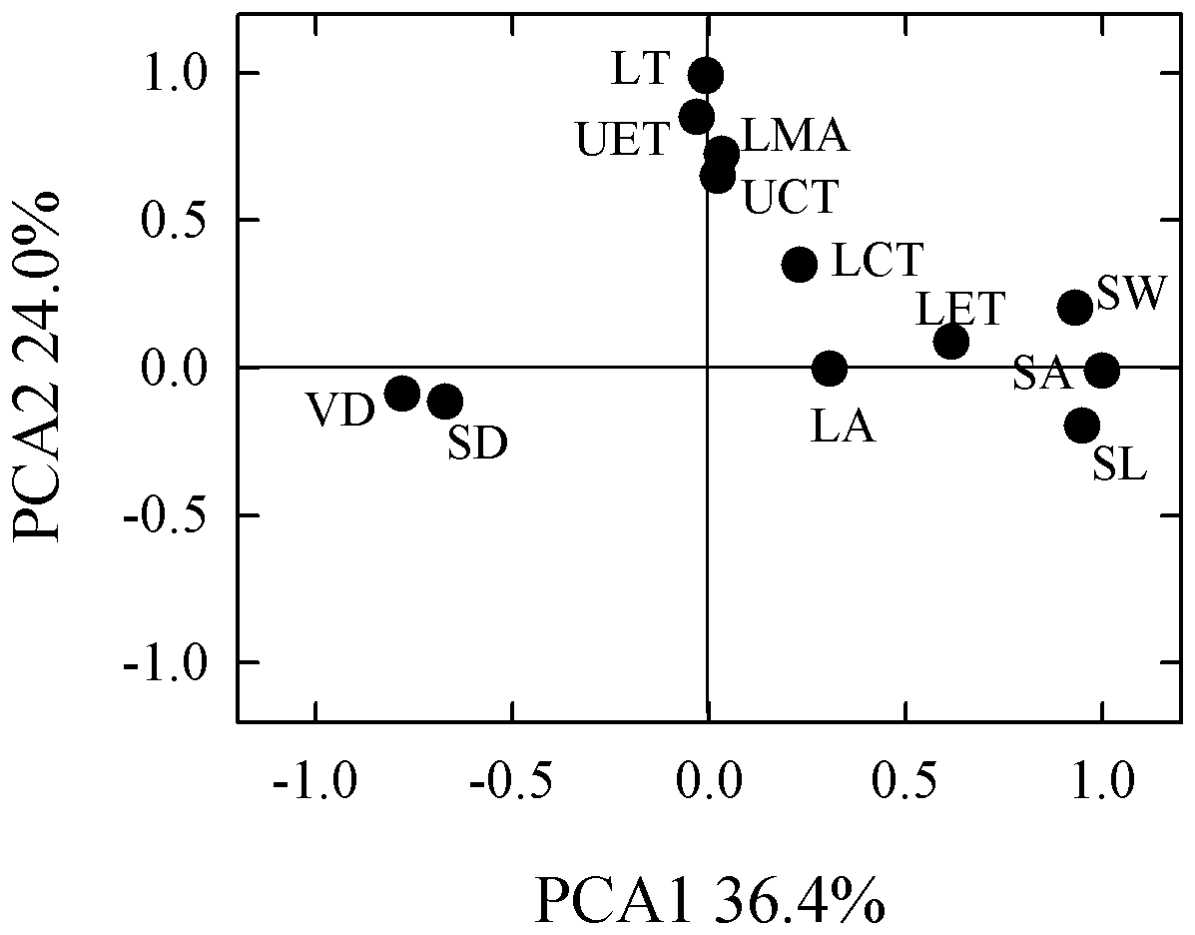
Factor-loading for stomatal and leaf traits along 2 axes of principal component analysis (PCA). SL, stomatal length; SW, stomatal width; SA, stomatal area; SD, stomatal density; UET, upper epidermal thickness; UCT, upper cuticular thickness; LET, lower epidermal thickness; LCT, lower cuticular thickness; LMA, leaf mass per unit area; LA, leaf area; VD, vein density; and MT, mesophyll thickness.

**Figure 4:**
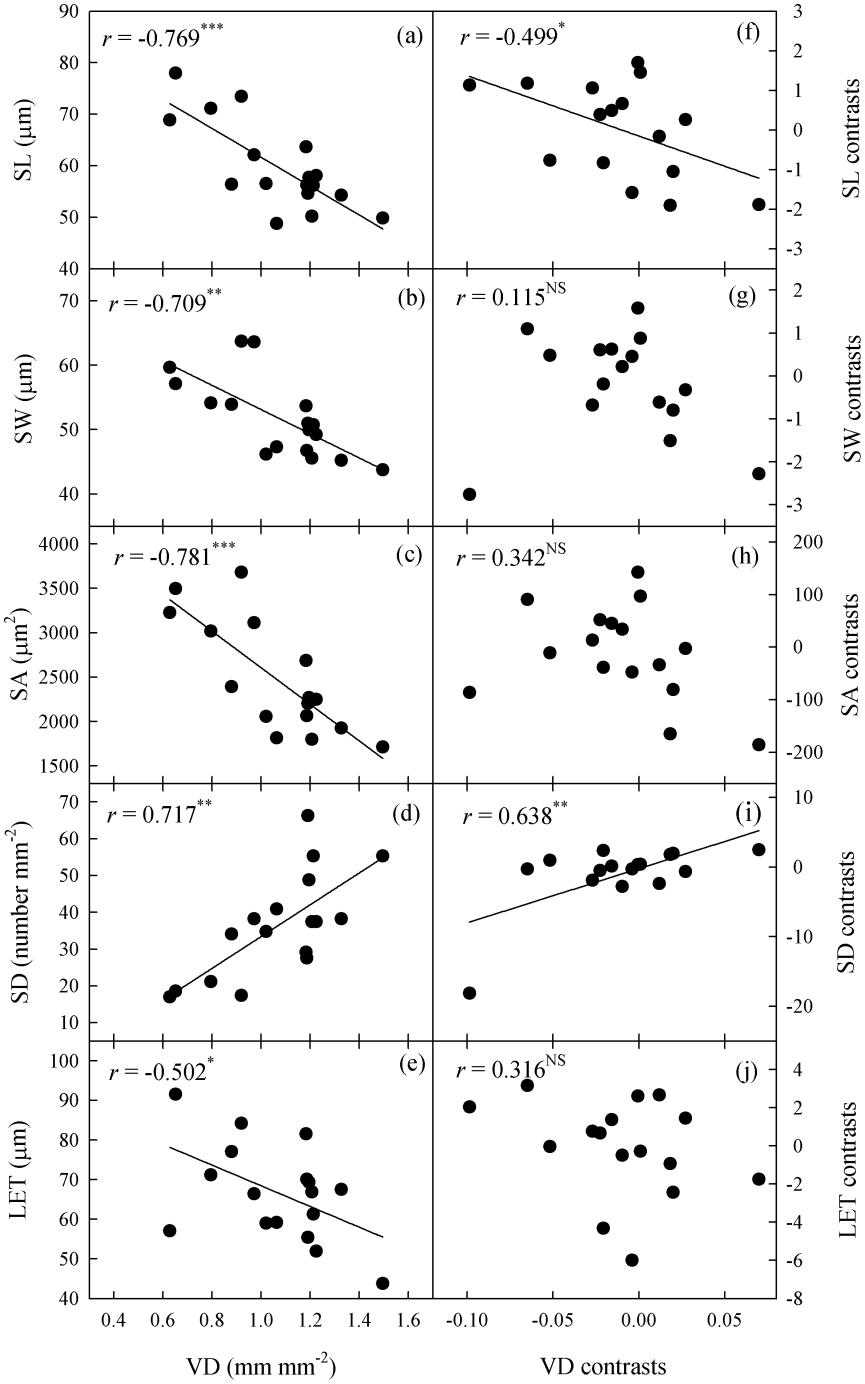
Correlations vein density with stomatal traits or lower epidermal thickness. Plate (a) to (e), Pearson’s regressions; and plate (f) to (j), phylogenetically independent contrast correlations. VD, leaf vein density; SL, stomatal length; SW, stomatal width; SA, stomatal area; SD, stomatal density; and LET, lower epidermal thickness.

**Figure 5:**
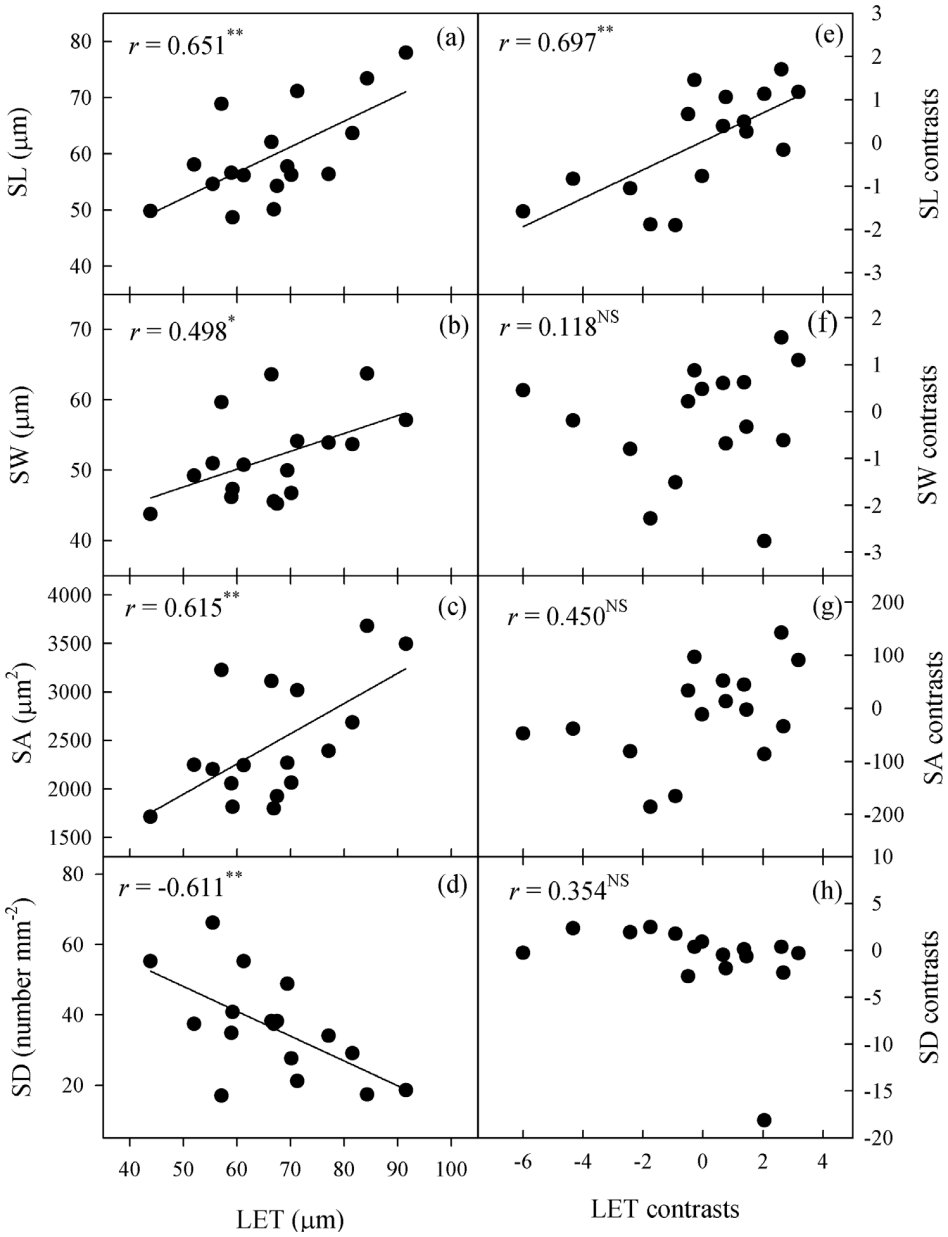
Correlations of lower epidermal thickness (LET) with stomatal traits. Plate (a) to (d), Pearson’s regressions; and plate (e) to (h), phylogenetically independent contrast correlations. SL, stomatal length; SW, stomatal width; SA, stomatal area; and SD, stomatal density.

### Histological Observations

From samples of all 17 species, the middle portions of mature leaves were fixed in FAA (formalin, glacial acetic acid, ethanol, and distilled water; 10:5:50:35, v:v:v:v) for at least 24 h. They were then dehydrated in an ethanol series and embedded in paraffin for sectioning. Transverse sections, made on a Leica RM2126RT rotary microtome (Leica Inc., Bensheim, Germany), were mounted on glass slides. These tissues were examined and photographed under an Olympus U-CMAD3 light microscope. Thicknesses of the upper cuticle (UCT, µm), upper epidermis (UET, µm), palisade tissue (PTT, µm), spongy tissue (STT, µm), lower epidermis (LET, µm) and lower cuticle (LCT, µm) were measured at the midpoint of each transverse section with Adobe Photoshop 8.0 (Adobe Systems Inc., California, USA). For each species, six leaves were taken from different plants.

**Figure 6:**
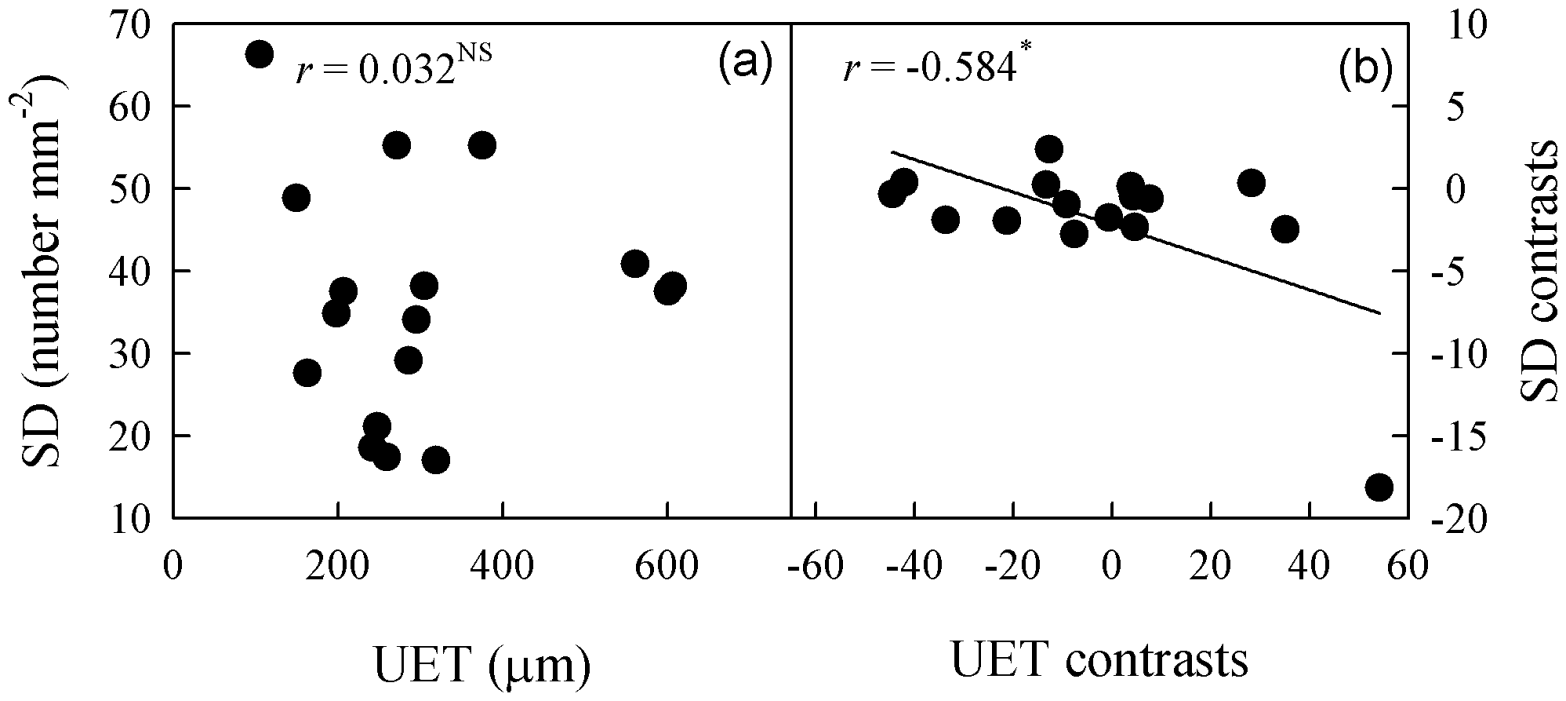
Correlation of upper epidermal thickness (UET) with stomatal density (SD). (a) Pearson’s regression, and (b) phylogenetically independent contrast correlation.

**Figure 7:**
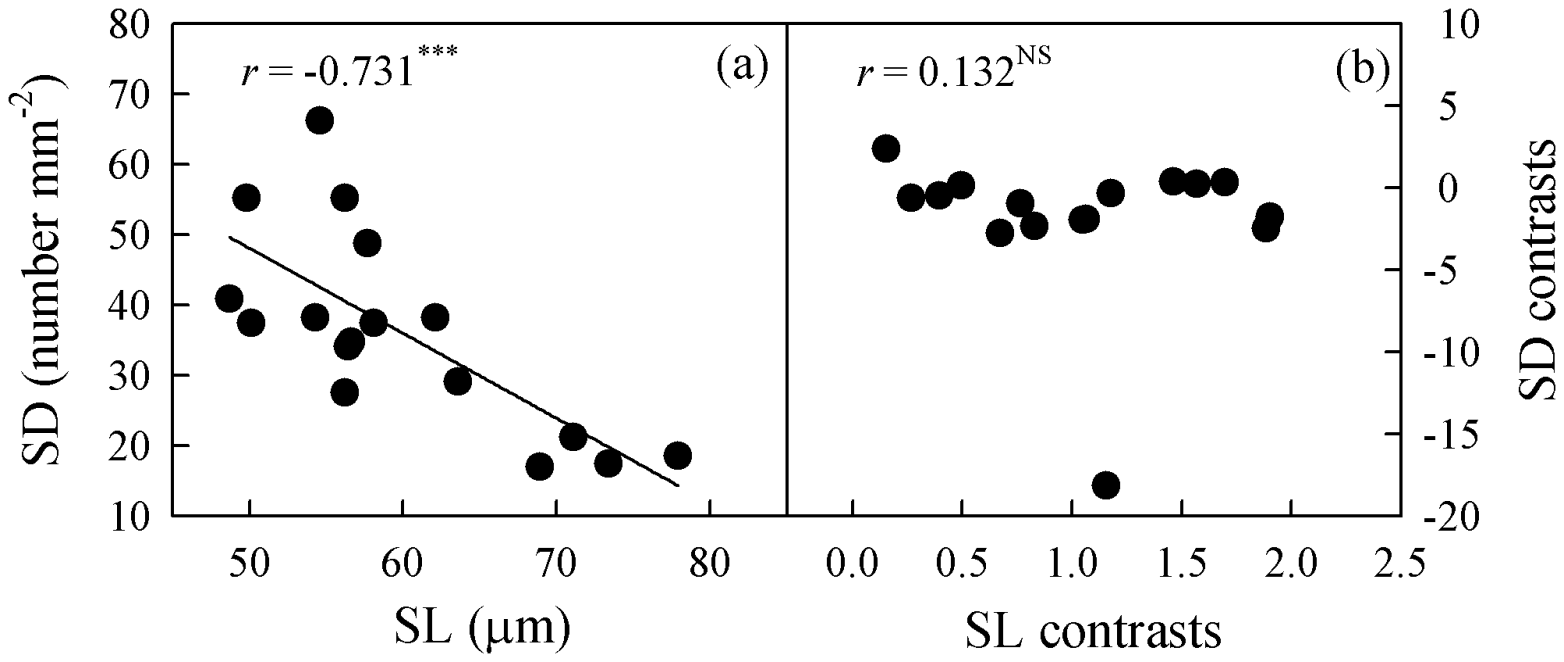
Correlation of stomatal length (SL) with stomatal density (SD) . (a) Pearson’s regression, and (b) phylogenetically independent contrast correlation.

### Stomatal Observations

The adaxial and abaxial epidermises were peeled from the middle portions of fresh, mature leaves, and images were made under an Olympus U-CMAD3 light microscope. For each species, six leaves from different plants were used for stomatal observations. Their stomata were tallied in 30 randomly selected fields. Stomatal density (SD) was calculated as the number per unit leaf area. Stomatal size was represented as the guard cell length, possibly indicating the maximum potential opening of the pore [43]. Stomatal length (SL, µm) and stomatal width (SW, µm) were measured from 30 stomata selected randomly. Stomatal area (SA) was calculated as 1/4 × π × SL × SW [44]. Stomatal index (SI) was estimated as the ratio of stomatal numbers per given area divided by the total number of stomata and other epidermal cells within the same area.

### Data Analysis

A phylogenetic signal (*K*) can be used to express the conservatism of traits. Cases where *K*<1 indicate convergent traits, *K* = 1 implies that closely related species have trait values that completely agree with a Brownian model, and *K*>1 represents traits more conserved than presumed from a Brownian expectation [45]. Our phylogenetic tree of *Paphiopedilum*, based on nuclear rDNA ITS sequences, was obtained from a previous report by Cox *et al*. [46]. The *K* value for each trait was calculated using ‘picante’, based on the R package 2.14 [47].

A principal component analysis (PCA) was performed with the ‘prcomp’ function of the R package ‘vegan’ to characterize the associations among leaf attributes and stomatal traits. Relationships among variables were analyzed using both Pearson regressions in R package 2.14 and phylogenetically independent contrasts (PICs). Possible evolutionary associations were assessed via PIC analysis, utilizing molecular phylogenetic trees [46]. This PIC analysis was evaluated with the “analysis of traits” (AOT) module in Phylocom, a program that calculates the internal node values for continuous traits [48], [49].

## Results

None of the species tested within *Paphiopedilum* had pubescent leaves, and all were hypostomatic. Although leaf and stomatal traits varied considerably across species (Tables 1, 2, Fig. 1), the magnitudes of variation were generally smaller for the stomata. Among species, fluctuations in SL, SW, SI, LCT and TCT were less than 2.0-fold, while those in VD, LMA, LA, SA, SD, UET, UCT, PTT, STT, LET and MT differed by 2.1- to 5.7-fold. For stomatal traits, the magnitude of variation was largest for SD (3.9-fold) and smallest for SW (1.4-fold). For leaf attributes, UET exhibited the largest variation (5.7-fold) across species while LCT showed the smallest range. The stable carbon isotope ratio (δ^13^C) ranged from –27.24‰ to 23.32‰ (Table 1). Values for SD and VD differed significantly among growth habits, whereas the other traits showed no significant differences. Both SD and VD tended to increase from terrestrial to facultative and epiphytic orchids (Fig. 2).

All stomatal traits (SL, SW, SA and SD), plus VD, LET, and LA, loaded mainly on the first PCA axis, explaining 36.4% of the total variation (Fig. 3). By contrast, SD and VD loaded in the opposite direction on that axis. Leaf attributes, including LT, LMA, UET, MT, UCT, and LCT, loaded on the second axis, explaining 24.0% of the total.

Vein density was correlated with SL, SW, SA, SD, and LET; after phylogeny was considered, VD was still correlated with SL and SD (Fig. 4). Values for LET were correlated positively with SL, SW and SA, but negatively with SD (Fig. 5). After eliminating any phylogenetic effects via PICs, those correlations of LET with SW, SA, and SD became insignificant. Stomatal index was not correlated with any leaf structural straits.

The UET was not correlated with SD when phylogeny was not considered, but a significant correlation was found between them after phylogenetic correction (Fig. 6). Conversely, stomatal density was positively correlated with stomatal length when a Pearson regression was used, but that correlation became insignificant after correction (Fig. 7). Neither leaf size nor thickness was correlated with SD or VD under any circumstances.

## Discussion

The evolutionary coordination of stomatal density with leaf thickness has been assessed in numerous species [8]. Here, we took a phylogenetically comparative approach to examine the correlated evolution between stomatal traits and leaf attributes from closely related species of *Paphiopedilum* grown under controlled conditions. Vein density had an evolutionary association with stomatal density and size, but traits for stomata and leaf thickness showed independent evolution.

### Leaf Attributes and Stomatal Traits in *Paphiopedilum*

Leaves were fleshy and had cuticles on both sides. These characters are common among xeromorphic plants. Growth habit had no obvious influence on LMA, LT or cuticle thickness (Fig. 2). Samples from all species were hypostomatic, and their stomata were sunken into the leaf epidermis. This adaptive feature shields exerophytic plants from the effects of desiccating winds, and can help prevent excessive transpiration losses [50]. Compared with data reported from other angiosperms, *Paphiopedilum* members had relatively lower VD and SD, but larger stomata [9], [51]. In fact, previous study has suggested that the species in Orchidaceae have, relatively, the lowest SD values in the entire plant kingdom [40]. We noted that epiphytic *Paphiopedilum* had higher VD and SD than the terrestrial species (Fig. 2). Dunbar-Co *et al.* have also found that taxa in *Plantago* growing on drier sites have higher VD [9]. As a whole, these leaf attributes and stomatal traits reflect a general trend in how land plants adapt when water is limited.

### Relationship of Leaf Attributes and Stomatal Traits to Phylogeny

Traits for both leaf anatomy and stomata varied significantly across species, although to a lesser extent for the latter (Table 1, Fig. 1). Several traits, such as SL, SA, UCT and TCT, showed strong phylogenetic signals while SD and SI exhibited a strong convergent evolution. This high level of conservatism demonstrates a distinct evolutionary shift among species [1]. Somewhat contradictory to our findings, Beaulieu *et al*. [43] did not report strong signals in SL (*K* = 0.685) or SD (*K* = 0.540) for 101 angiosperm species. However, Hodgson *et al*. [20] noted that stomatal size was related to both cytological status and phylogeny. The discrepancy between our observations and those of Beaulieu *et al*. are probably related to the choice of plant materials tested. In that earlier study, three growth forms were selected (herb, tree, and shrub), which led to large genetic differences. By contrast, our examination utilized tissues from the same genus, with all plants exposed to the same greenhouse conditions and, consequently, revealing only small genetic differences.

The strong signals for SL, SA, UCT, and TCT indicated that those traits are phylogenetically conserved. However, most traits had weak signals, possibly because of a departure from Brownian motion evolution, such as adaptive evolution, that would not have been correlated with phylogeny. Therefore, this reflected the outcome of selection in heterogeneous environments where species can best acclimate to their current growing conditions [1]. Caruso *et al.* [52] have suggested that any constraints on the development of stomatal traits in *Lobelia cardinalis* primarily arise from a lack of genetic variation. In our study, the correlation between LET and either SD or SA disappeared when the effect of phylogeny was considered, thus confirming that variations in stomatal traits and leaf attributes are related to that particular influence.

### Evolutionary Associations of Stomatal Anatomy with Leaf Traits

Vein density in *Paphiopedilum* was positively correlated with stomatal density, whether or not phylogeny was considered. However, VD was negatively correlated with stomatal size (Fig. 4), indicating that leaf vein has an evolutionary association with stomatal anatomy. This result supports the notion that the development and function of leaf veins and stomata are coordinated [11], as the coordinated development of veins and stomata is important for optimizing photosynthetic yield relative to carbon investment in leaf venation [11]. Moreover, coordinated plasticity in veins and stomata is thought to be at least partially related to leaf size; the development of leaf-size plasticity can provide an efficient way for plants to acclimate their hydraulic and stomatal conductance to contrasting transpirational demands under different lighting conditions [11], [51]. However, we found that SD and VD for these 17 *Paphiopedilum* species were not affected by leaf size. This was because our experimental materials had been grown in the same environment, and had similar transpirational demands.

We found no evidence for correlated evolution between stomatal traits and leaf thickness or cuticle thickness, which suggests a lack of functional association. Although LET was correlated with stomatal traits when phylogeny was not considered, only two correlations (LET vs SL, UET vs SD) were significant after that correction. The discrepancy between our Pearson’s and PIC correlations can be explained in that PICs reflect the historical pattern of diversification among taxa, whereas traditional Pearson’s correlations describe present-day relations among taxa [1]. Similar to our results, Beerling and Kelly [8] have suggested that thicker leaves do not necessarily mean more stomata. Nevertheless, previous studies have also shown that species with thick leaves have moderately large stomata [20], and that leaf thickness is negatively correlated with SD along an acidity gradient [18].

The lack of evolutionary correlation of stomatal traits with leaf thickness or cuticle thickness may have several explanations. Selective pressure that drives their development can differ between the two. Evolutionary trends largely depend on the selective force endured in challenging environments [9]. Stomatal density can be influenced by atmospheric CO_2_ concentration, heat stress, water status, plant density and light intensity [13], [16], [17], whereas leaf thickness is affected by light intensity, UV-radiation, rainfall and the supply of soil nutrients [31], [35], [38]. This inconsistency in evolutionary correlations among functional traits suggests that fundamentally different selective pressures and constraints may be acting [53]. Consequently, for the genus studied here, periodic water shortages and low nutrient availability in karst regions would have contributed to the evolution of leaf anatomy.

The difference in function between leaf cuticle thickness and stomatal traits decreases the coordination between them. In fact, changes in leaf anatomy do not always reflect adaptations to water availability. For example, leaves of plants growing in habitats with reduced soil nutrients have thicker epidermises than do their relatives in high-nutrient soils [30] because those sclerophyllous tissues develop as a way to protect scarce nutrient investments in leaf material against herbivory and abiotic physical damage [37]. By contrast, in arid environments, a thick cuticle likely has other functions besides that of water barrier, such as preventing physical damage by herbivorous pests [54].

The structural investment toward different leaf traits is largely controlled by an evolutionary trade-off between the antagonistic demands to maximize both photosynthesis and WUE [19], [55]. Having a thicker cuticle implies a greater construction cost for the leaf protective structure [28]. If more biomass must be allocated to the same function, the investment is reduced toward other functions. This situation is not cost-efficient to plant survival and competitiveness. Therefore, a correlated evolution among those traits would limit such divergence and adaptive selection [1]. Although many leaf surface characters, e.g., crypts, wax and hairs, can modify the relationship between stomatal size and number, and stomatal function, an evolutionary association between leaf anatomical traits and stomatal traits does not always necessitate water conservation and ecological strategies.

### Correlation between Stomatal Density and Size

Stomatal density was significantly correlated with SL, but that association disappeared when phylogeny was considered. The negative correlation found here between SD and SL has been described previously [43], [56]. Both stomatal aperture and density are linked to leaf conductance, photosynthetic carbon gain and transpiration [55]. The capacity of plants to fix carbon is constrained by their photosynthetic biochemistry and CO_2_ diffusion conductance. When the concentration of atmosphere CO_2_ decreases, stomata become denser while the rate of maximum Rubisco carboxylation (V_cmax_) slows. This co-variation among SL, SD and the V_cmax_ rate reduces the impact that any change in atmospheric CO_2_ has on the assimilation of leaf CO_2_, resulting in minimum energy cost and reduced nitrogen requirements [3]. A negative correlation between SD and SL also increases plasticity in maximum stomatal conductance to water vapor and CO_2_, with minimal alterations in the balance of water loss and epidermal allocations to the stomata [14], [56].

In summary, phylogeny has a significant effect on leaf traits and stomatal traits in *Paphiopedilum.* Stomatal length and area and upper cuticle thickness are strongly conserved. We noted a correlated evolution between stomatal traits and vein density in *Paphiopedilum*, but not between stomatal traits and leaf thickness. These findings provide insight into the development of traits related to water economy by orchids under natural selection.
